# Reproductive genome editing interventions are therapeutic, sometimes

**DOI:** 10.1111/bioe.12846

**Published:** 2021-02-07

**Authors:** César Palacios‐González

**Affiliations:** ^1^ University of Oxford Uehiro Centre for Practical Ethics ‐ Faculty of Philosophy Oxford United Kingdom of Great Britain and Northern Ireland

**Keywords:** assisted reproduction, CRISPR, gene editing, genome editing, germline editing, therapy

## Abstract

In this paper I argue that some human reproductive genome editing interventions can be therapeutic in nature, and thus that it is false that all such interventions just create healthy individuals. I do this by showing that the conditions established by a therapy definition are met by certain reproductive genome editing interventions. I then defend this position against two objections: (a) reproductive genome editing interventions do not attain one of the two conditions for something to be a therapy, and (b) some reproductive genome editing interventions are therapeutic but in a nonstandard way. In the Conclusion I call for a more nuanced discussion of the nature of reproductive genome editing interventions.

## 1 INTRODUCTION

In a recent paper Tina Rulli has argued that reproductive uses of CRISPR‐Cas9 are not therapeutic in nature: ‘reproductive uses of CRISPR do not save lives, cure, or offer a unique opportunity for disease prevention’.^1^ Even when she limits her discussion to reproductive uses of CRISPR‐Cas9 (henceforth rCRISPR) her argument applies to all current reproductive genome editing techniques (e.g. TALEN and ZFNs). Rulli’s stance on the non‐therapeutic nature of reproductive genome editing interventions is shared by several authors; most recently, for example, by Peter Mills and Owen Schaefer.^2^ If Rulli were correct then what is thought to be one of the strongest arguments for the development and implementation of reproductive genome editing interventions would fail. It would do so because it is grounded on the assumption that these interventions are therapeutic in nature. For example, Julian Savulescu et al. maintain that:

There is a moral imperative to continue this research [in human embryos]. Gene editing technologies have enormous potential as a therapeutic tool in the fight against disease […] Advanced and precise gene editing techniques could virtually eradicate genetic birth defects, thereby benefiting nearly 8 million children every year.^3^

Before moving forward, let me define what are reproductive genome editing interventions. Reproductive genome editing interventions are a subset of genome editing interventions that are carried out in gametes, gamete progenitor cells, and embryos. These changes in the genome can be passed down to future generations. In addition to reproductive genome editing interventions we have *somatic genome editing interventions*. Somatic genome editing interventions are those which cannot be passed down to future generations, their effects are limited to the individuals who undergo the interventions. The present paper will not discuss somatic genome editing interventions.

Rulli presents her case against reproductive genome editing interventions being therapeutic in the following way. First, she advances a definition of what ‘cures or therapies’ are. Second, she shows that reproductive genome editing interventions do not meet one of the necessary conditions stipulated in the definition. Finally, she concludes that the use of such biotechnologies is not therapeutic in nature. Rulli employs this conclusion to further argue that the moral reasons that we have to develop and invest in such biotechnologies are weaker than first thought. And that when confronted with resource allocation decisions we should favour the development of *actual* therapies over biotechnologies that only create certain types of individuals (i.e. healthy individuals).

In this paper I argue, contra Rulli, that some reproductive genome editing interventions can be therapeutic, and thus that it is false that all such interventions just create healthy individuals. It is important to bear three things in mind. First, my main goal is to answer a question about the *nature* of reproductive genome editing interventions: Can reproductive genome editing interventions be therapeutic? Second, I am not trying to answer a statistical question about the real world uses of such technologies: How often are reproductive genome editing interventions therapeutic, if indeed they are? Third, I am not engaging with the question of what type of moral reasons there are in favour, or against, of offering reproductive genome editing interventions if indeed they are therapeutic.^4^ I am not doing so since here I do not wish to mix the metaphysical discussion with the ethical one.

The paper proceeds as follows. In the next section, I present Rulli’s case against reproductive genome editing interventions being therapeutic. In the third section, I discuss why it is important to differentiate between (a) the clinical decision to employ a reproductive genome editing intervention, and (b) the actual editing process. In the fourth section, I rely on the distinction presented in the third section to show that the nature of some reproductive genome editing interventions is therapeutic, and therefore that Rulli’s case fails. In the fifth section, I argue against the position that reproductive genome editing interventions are therapeutic but in a nonstandard way. In the Conclusion, I take stock of the arguments advanced in the paper, and call for a more nuanced discussion of the nature of reproductive genome editing interventions.

## 2 REPRODUCTIVE GENOME EDITING INTERVENTIONS ARE NOT THERAPEUTIC

Any discussion on whether reproductive genome editing interventions are therapeutic must start by addressing the issue of what makes an intervention a therapy or cure. Rulli defines ‘treatment or cure’ in the following way:

For an intervention X to count as a treatment or cure, in addition to it being the case that (a) if X is administered, it will help soothe, heal, or remedy someone’s illness, it is also the case that (b) if X is not administered, a person will suffer more or die earlier than if it had been.^5^

Let us accept, for the sake of argument, this definition. There are two important things to note here. First, (b) is a counterfactual condition. Second, Rulli maintains that the definition does not allow room for uncertainty. Given this she revises her account in the following way:

We should understand that an intervention X counts as a treatment if its administration decreases to *a significant degree* the likelihood of person P getting disease D in the case that it is administered compared with the case where it is not administered.^6^

One obvious issue with the revised definition (and the original one) is that it is under‐inclusive. It does not take into consideration that interventions carried out in non‐persons, for example dogs, can be therapeutic. In order to deal with this issue, let us simply replace ‘person’ with ‘organism’^7^ in the two previous definitions. Once we have defined ‘treatment’ or ‘cure’ in such a way we can investigate if reproductive genome editing interventions (i.e. the genome editing of gametes, gamete progenitor cells, and embryos) meet the conditions specified in the definition(s).

According to Rulli the first step we need to take for doing so is to list the options available to prospective parents that are at risk of transmitting a genetic disease and can resort to rCRISPR. These are:

1. Create a child in a genetically modified and healthy state using rCRISPR.
2. Create a child with a substantial risk of some genetic disease D, not using rCRISPR.
3. Do not create a child at all.^8^

Rulli notes that there is an asymmetry in the number of available general courses of action that such prospective parents have, and the number of available general courses of action that people suffering from medical *non‐reproductive* conditions have. In medical *non‐reproductive* scenarios an individual considering a therapy could choose between: (a) partaking in therapy, or (b) refraining from partaking in therapy. For example, someone with bacterial pneumonia could: decide to take an antibiotic, or decide to do nothing. Importantly, not choosing partaking in therapy entails (b) as default. Reproductive genome editing scenarios are different, according to Rulli. Refraining from the first option (i.e. creating a child in a genetically modified and healthy state using rCRISPR) does not default in the second option (i.e. creating a child with a substantial risk of some genetic disease D).

According to Rulli, prospective parents in reproductive genome editing scenarios can choose either (2) or (3), after (1) has been rejected. The existence of the possibility of choosing between (2) and (3) makes Rulli conclude that reproductive genome editing interventions do not satisfy the counterfactual condition (b) of her first therapy definition. It is not the case that if rCRISPR, for example, *is not* carried out a human organism will suffer more or die earlier than if rCRISPR *is* carried out. She maintains this because: ‘In the rCRISPR case, the existence of a child, with or without disease, is not inevitable—i.e., her existence is still a matter of distinct and separate choice’^9^ and ‘It is both a metaphysical and moral stretch to call a not‐yet‐created or unimplanted embryo a patient, since her existence is not a given—it is the very thing under consideration and under our control’.^10^ That reproductive genome editing interventions do not satisfy the counterfactual condition (b), of the first definition, makes Rulli conclude that they are not therapeutic. The argument can be presented in the following way:

For an intervention X to count as a treatment or cure, in addition to it being the case that (a) if X is administered, it will help soothe, heal, or remedy someone’s illness, it is also the case that (b) if X is not administered, an organism will suffer more or die earlier than if it had been.
If prospective parents decide not to carry out reproductive genome editing interventions no individuals will suffer more or die earlier because of such decisions, given that no individuals that could be affected by such interventions exist at the point when the decisions are made and their existence is not inevitable.

From (1) and (2).

Reproductive genome editing interventions are not therapeutic.

## 3 THE ACTUAL EDITING PROCESS AND THE CLINICAL DECISION TO EMPLOY

The first thing we need to do in order to understand why Rulli’s argument is flawed—and that reproductive genome editing interventions *can* be therapeutic—is to pay attention to the difference between the *actual editing process* and the *clinical decision to employ* a genome editing technology.^11^ Let us begin by examining the actual editing process. Genome editing of *embryos* can affect either numerical identity or qualitative identity. In a genome editing intervention that affects qualitative identity, embryo A retains its numerical identity but one of its qualities is changed. In a genome editing intervention that affects numerical identity, embryo A ceases to exist and a new embryo, B, comes into existence.

Here is an uncontroversial example of what would be a numerical identity affecting genome editing intervention in an embryo. Embryo A would cease to exist—and embryo B would come into existence—if it were the case that A’s sexual chromosome ‘Y’ was edited out in its entirety, and a sexual chromosome ‘X’ was edited in.^12^ Whether a genome editing intervention on an embryo affects numerical or qualitative identity will depend, partly, on the extent of the editing. For our current purposes we do not need to present a detailed account of when an editing procedure is numerical (or qualitative) identity affecting, we only require that there be genome editing procedures that are so; and this seems to be clearly the case as per the previous example.^13^

Let us now examine the *clinical decision* to employ a reproductive genome editing technology. On the one hand, the clinical decision to employ rCRISPR can occur *prior* to the creation of the embryo that will be subject to such intervention. Imagine a couple where one of the prospective parents has known since childhood that he is homozygous for a dominant genetic disease of late‐onset. The couple decides to go to a fertility specialist, since they want to have a healthy child that is genetically related to both of them. The fertility specialist tells them that they can choose to undergo IVF to create some embryos and that afterwards she can use CRISPR to correct the deleterious genetic mutations in all of them. Importantly, if the couple decides to follow the CRISPR path laid down by the doctor then this decision affects which embryos will be brought into existence; because the medical protocol for inducing ovulation and sperm collection affects which sperm and which eggs will be brought together. Here I am accepting the position known as the Origin View. As Parfit describes it, this is the view that ‘each person has this distinctive necessary property: that of having grown from the particular pair of cells from which this person in fact grew’.^14^

On the other hand, the clinical decision to employ CRISPR can be made *after* the creation of the embryo that will be subject to such intervention. Imagine that a couple undergoes IVF and creates some embryos, which are then cryopreserved. A couple of months after the IVF procedure has taken place the prospective mother is informed that she is homozygous for a dominant monogenetic disease of late‐onset. The couple visits their fertility doctor, who tells them not to worry since they can decide to employ CRISPR in order to correct the deleterious genetic mutations of their already stored embryos. Here the decision to employ CRISPR is not among the causes of the decision to create the embryos. Importantly, this type of case shows that Rulli’s third option (i.e. do not create a child at all) is not open when deciding whether to carry out certain reproductive genome editing interventions.^15^ This is relevant because she maintains that ‘It is the existence of option (3) that grounds the fundamental difference between rCRISPR and conventional treatments’.^16^

To sum up: the actual genome editing process can be either numerical identity affecting or qualitative identity affecting; and the clinical decision to employ a genome editing technology can be numerical identity determining (i.e. it specifies which embryo will be created) or not.

Now, since gametes and gamete progenitor cells are not human organisms then we must conclude that any in vitro genome editing carried out in them would not be therapeutic for an *existing* human organism. This is true regardless of whether the intervention is therapeutic for the cells.^17^ Finally, in the previous section we noticed that Rulli maintains that it is a metaphysical stretch to call an unimplanted embryo a patient since the embryo’s existence is not a given.^18^ This assertion is false for two reasons. First, the human embryo’s existence, as a human organism, is evident; and this is so irrespective of the embryo’s future lifespan or the fact that it is a person or not.^19^ Second, all organisms can be patients, in the sense that they can be the subject of interventions that would fulfil both conditions of the first therapy definition.

## 4 TO CURE OR TO CREATE, THAT IS THE QUESTION

Rulli accepts that those cases where genome editing is carried out on existing embryos are problematic for her conclusion (i.e. reproductive genome editing interventions are not therapeutic). The problem stems from the fact that these embryos are, in Rulli’s nomenclature, inevitable. The numerical identity of any existing unimplanted embryo, as a human organism, is already established when the genome editing intervention is carried out.^20^ Therefore, one of the objections that Rulli tries to address is: ‘At least in the cases where rCRISPR is actually used directly on embryos with genetic defects, then it is actually a therapy’.^21^

One thing to note is that the objection, as stated, fails, but not for the reason that Rulli maintains, and which I will explore later on. The objection fails because a reproductive genome editing intervention can affect the embryo’s numerical identity. In these cases such a genome editing intervention *is not therapeutic* since neither condition of Rulli’s first therapy account would be attained. The editing would not remedy an individual’s illness, since it would create a different individual. And it is not the case that if the editing had not happened the new individual would suffer more and die earlier than if it had happened, since if the genome editing intervention had not happened then this new individual would not have been created.

Here is a revised version of the objection that Rulli must confront: where rCRISPR is actually used directly on embryos with genetic defects, *and it only affects their qualitative identity*, then it is actually a therapy.^22^ This revised objection also fails. It does so because identity preserving genome editing interventions could *harm* the embryo, all things considered. In other words, there are identity preserving genome editing interventions that are not therapeutic. This being so I have revised again the objection: where rCRISPR is used directly on embryos with genetic defects, and it only affects their qualitative identity, then it *could* actually be a therapy. We can now explore why this objection is problematic for Rulli.

There is a subset of cases where the reproductive genome editing intervention only affects the embryo’s qualitative identity that show that Rulli’s argument fails. These are those that fulfil both conditions of her first therapy definition. For example, if through genome editing we replaced the gene that causes the monogenetic disease of Huntington’s^23^ then we would only affect the embryo’s qualitative identity. This means that embryo A continues to be embryo A after the editing procedure. It is not the case that embryo A has ceased to exist and that embryo B has been brought into existence. Further, the genome intervention in this scenario is therapeutic because:

- if the genome editing is carried out it will help remedy an individual’s (future) illness, and
- if the genome editing did not happen an individual would (in the future) suffer more or die earlier than if it had happened.

Someone could argue that here we did not engage with Rulli’s revised account of therapy, and consequently that our argument (i.e. genome editing interventions *can be* therapeutic) fails to meet its target. However, such a procedure would count as a therapy on the revised account because:

- carrying out the genome editing intervention decreases to a significant degree the likelihood of individual Z getting Huntington’s, compared with the case where the editing is not carried out.

This case clearly shows that the *nature* of *some* reproductive genome editing interventions *is* therapeutic. How does Rulli defend against this objection? She does not. Rulli accepts that the genome editing of embryos—that have a deleterious genetic mutation—can be therapeutic ‘in the most technical sense’.^24^ Finally, let us remember that we are not trying to answer a statistical question (i.e. how often are reproductive genome editing interventions therapeutic), were are trying to answer a metaphysical one.

## 5 ARE REPRODUCTIVE GENOME EDITING INTERVENTIONS THERAPEUTIC IN A NONSTANDARD WAY?

Rulli goes on to argue that there is a very significant difference between a case like the one I presented (i.e. Huntington’s) and what she considers to be *standard* therapeutic interventions. The difference, according to her, lies in that ‘we only create an embryo with defects in the first place so that we can then manipulate it using CRISPR’.^25^ From this Rulli moves on to suggest that even if the actual editing process is therapeutic its moral value is suspect, since the doctor’s action ‘*in its totality* [emphasis added] could hardly be called curative, for he first created the harm’.^26^ For her, rCRISPR type cases are morally analogous to a doctor first infecting a patient with a disease and then curing the patient. Rulli concludes that ‘If one insists on calling this a cure or a therapy it is certainly a nonstandard usage of those concepts; it warrants a distinction from standard cures that save lives and prevent otherwise probable harms’.^27^

It is important to note that Rulli here has pivoted from talking about the *nature of the actual editing process* to discussing the *clinical decision to employ* this type of intervention. She shifted from trying to show that reproductive genome editing interventions *are not* therapeutic, to trying to show that the *totality of actions* involved in some reproductive genome editing interventions *are therapeutic but in a nonstandard way*. Once we have clarified this we can proceed to examine the claim that: the totality of actions involved in a medically assisted reproductive process that includes a genome editing intervention in an unimplanted embryo are therapeutic but in a nonstandard way.

Rulli maintains that using the concept ‘therapy’ for reproductive genome editing interventions in unimplanted embryos entails using it in a nonstandard way because, as stated above, we first intentionally create an embryo with a certain genetic condition so that then we can go on and eliminate said condition. This claim is false for certain scenarios. Reproductive genome editing interventions can happen (as shown in a previous section) on unimplanted embryos whose creation antecedes any decision (or thought) about carrying out a genome editing intervention. We must thus conclude that reproductive genome editing interventions can be therapeutic in a standard way.

A second point is that Rulli has not proved that creating an individual with a genetic disorder harms the individual created. Rulli here is confronted with the Non‐Identity Problem. The Non‐Identity Problem, broadly speaking, asks the question of whether an action that results in the existence of an individual can harm that individual.^28^ In order for Rulli’s position on ‘nonstandard therapeutic actions’ to get off the ground she first needs to demonstrate that *harm* is caused to an individual by virtue of being brought into existence with a deleterious genetic mutation. Without this, we cannot conclude that the decision to intentionally bring into existence an individual with a deleterious genetic mutation is analogous to a doctor infecting a patient with a disease (i.e. a doctor causing harm to an individual) so later on she can cure her.

## 6 CONCLUSION

In this paper I have proved that some human reproductive genome editing interventions can be therapeutic in nature. I did so by showing that numerical identity preserving reproductive genome editing interventions in unimplanted embryos can attain the conditions established in a therapy definition. This shows that Rulli’s conclusion that rCRISPR ‘simply does not save or treat any lives at all; it only creates healthy ones where none was inevitable’^29^ is false. My conclusion stands even if it is the case that most reproductive genome editing interventions happen in gametes or gamete progenitor cells. It does so because the question we asked in the paper is a question about the *metaphysics* of such interventions, and not a question about *how often* they would be therapeutic. Here I have also shown that we need to be careful when discussing the nature of reproductive genome editing interventions. We need to clearly differentiate between numerical identity preserving and numerical identity affecting interventions, on the one hand, and between the clinical decision to employ an intervention and the actual intervention, on the other hand. Taking this more nuanced approach to discussing reproductive genome editing interventions can only benefit the debate.

## CONFLICT OF INTEREST

The author declares no conflict of interest.

## Footnotes

^1^

Rulli, T. (2019). Reproductive CRISPR does not cure disease. *Bioethics, 33*(9), 1072–1082. 10.1111/bioe.12663 31489967

^2^

Mills, P. F. (2020). Genome editing and human reproduction: The therapeutic fallacy and the ‘most unusual case’. *Perspectives in Biology and Medicine, 63*(1), 126–140. 10.1353/pbm.2020.0010; Schaefer, G. O. (2020). Can reproductive genetic manipulation save lives? *Medicine, Health Care and Philosophy*, *23*(3), 381–386. 10.1353/pbm.2020.0010 32063592

^3^

Savulescu, J., Pugh, J., Douglas, T., & Gyngell, C. (2015). The moral imperative to continue gene editing research on human embryos. *Protein & Cell.* 6(7), 476–479. 10.1007/s13238-015-0184-y PMC449105026113289

^4^

De Wert, G., Heindryckx, B., Pennings, G., Clarke, A., Eichenlaub‐Ritter, U., van El, C.G., … European Society of Human Genetics and the European Society of Human Reproduction and Embryology. (2018). Responsible innovation in human germline gene editing: Background document to the recommendations of ESHG and ESHRE. *European Journal of Human Genetics: EJHG, 26*(4), 450–470. 10.1038/s41431-017-0077-z; Nuffield Council on Bioethics. (2018). Genome editing and human reproduction: Social and ethical issues. London, UK: Nuffield Council on Bioethics. Retrieved from https://nuffieldbioethics.org/publications/genome‐editing‐and‐human‐reproduction; National Academies of Sciences, Engineering and Medicine. (2017). Human genome editing: Science, ethics, and governance. Washington, D.C: National Academies of Sciences, Engineering and Medicine. Retrieved from 10.1038/s41431-017-0077-z

^5^

Rulli, op. cit. note 1, p. 1076.

^6^

Ibid: 1077.

^7^

Here I am following Mathew Liao’s organism account: ‘Organisms are beings that have the capacities to carry on certain life processes. Some such processes may include metabolism, which is the capacity to break down substances and convert them to other substances that can be used by the body; growth, which is the capacity to increase the size of existing cells and the number of cells; assimilation, which is the capacity to absorb substances that are chemically different from those found in the body; responsiveness, which is the capacity to detect and respond to changes outside or inside the body; movement, which is the capacity to move the whole body, parts of the body such as organs, single cells, or even structures inside cells; and reproduction, which is the capacity to form new cells for growth, repair, or replacement or the formation of a new individual. Other life processes may include respiration, digestion, absorption, circulation, excretion, differentiation, and so on. Taxonomically, two kinds of organisms can be distinguished: unicellular and multicellular organisms’. Liao, S. M. (2006). The organism view defended. *The Monist, 89*(3), 334–350.

^8^

Rulli. op. cit. note 1, p. 1077.

^9^

Ibid. This position has also been defended by the Nuffield Council on Bioethics, op. cit. note 4, p. 22–23.

^10^

Rulli, op. cit. note 1, p. 1078. It must be noted that in their 1996 paper ‘Who benefits? — Why personal identity does not matter in a moral evaluation of germ‐line gene therapy’ Nils Holtug and Peter Sandøe had already identified this feature of reproductive genome editing technologies, when considering unimplanted embryos. Holtug, N., & Sandøe, P. (1996). Who benefits? — Why personal identity does not matter in a moral evaluation of germ‐line gene therapy. *Journal of Applied Philosophy, 13*(2), 157–166. 10.1111/j.1468-5930.1996.tb00158.x

^11^

Paying attention to this point is relevant when assessing if new biotechnologies are therapeutic or not. Because it can be the case that even if the *clinical decision* to employ X is numerical identity‐determining, the *actual process* X is numerical identity‐preserving. For a recent discussion on this topic, as applied to a different reproductive biotechnology, see: Palacios‐González, C. (2017). Are there moral differences between maternal spindle transfer and pronuclear transfer? *Medicine, Health Care and Philosophy, 20*(4), 503–5011. 10.1007/s11019-017-9772-3 PMC566596328429249

^12^

Wang, K., de la Torre, D., Robertson, W. E., & Chin, J. W. (2019). Programmed chromosome fission and fusion enable precise large‐scale genome rearrangement and assembly. *Science, 365*(6456), 922–926. 10.1126/science.aay0737 PMC705635531467221

^13^

There is vast literature, spanning at least 20 years, on whether gene editing affects identity: Cavaliere, G. (2018). Genome editing and assisted reproduction: Curing embryos, society or prospective parents? *Medicine, Health Care, and Philosophy, 21*(2), 215–225. 10.1007/s11019-017-9793-y; Delaney, J. J. (2011). Possible people, complaints, and the distinction between genetic planning and genetic engineering. *Journal of Medical Ethics, 37*(7), 410–414. 10.1007/s11019-017-9793-y; Elliot, R. (1997). Genetic therapy, person‐regarding reasons and the determination of identity. *Bioethics, 11*(2), 151–160. 10.1007/s11019-017-9793-y; Persson, I. (1997). Genetic therapy, person‐regarding reasons and the determination of identity — a reply to Robert Elliot. *Bioethics, 11*(2), 161–169. 10.1007/s11019-017-9793-y; Holtug & Sandøe, op. cit. note 10, 157–166; Persson, I. (1995). Genetic therapy, identity and the person‐regarding reasons. *Bioethics, 9*(1), 16–31; Elliot, R. (1993). Identity and the ethics of gene therapy. *Bioethics, 7*(1), 27–40. 10.1007/s11019-017-9793-y; Kahn, J. P. (1991). Commentary on Zohar’s "Prospects for ‘genetic therapy’ ‐‐ can a person benefit from being altered?" *Bioethics, 5*(4), 312–317. 10.1007/s11019-017-9793-y; Kahn, J. P. (1991). Genetic harm: Bitten by the body that keeps you? *Bioethics, 5*(4), 289–308. 10.1007/s11019-017-9793-y; Zohar, N. J. (1991). Prospects for ‘genetic therapy’ ‐‐ can a person benefit from being altered? *Bioethics, 5*(4), 275–288. 10.1007/s11019-017-9793-y

^14^

Parfit, D. (1984). *Reasons and persons*. UK: Oxford University Press. Even though Rulli does not explicitly mention the Origin View in her paper, she implicitly accepts it when she discusses the Non‐Identity Problem: 'To be clear: the argument here is distinct from the concern about the Non‐Identity Problem, where the choice of whether to use a certain technology changes the gametes used to create a child and thus changes the identity of the child who comes to exist'. Rulli, op. cit. note 1, p. 1077.

^15^

It could be objected that the embryo and the child are not one and the same individual (i.e. they are numerically distinct). And thus even if the gene editing intervention could be therapeutic for the embryo it would not be so for the child, since the child comes into existence at a later point. Under the organism view (op. cit. note 7) this particular intervention would not alter the numerical identity of the embryo, and thus the embryo and the child are one and the same individual but at different times. However, under the psychological view the embryo and the child are distinct individuals. Now, my objection to Rulli holds here because she and I are departing from the organism view when discussing whether genome editing interventions can be therapeutic for embryos.

^16^

Rulli, op. cit. note 1, p. 1077.

^17^

There might be very rare cases where (a) we have preselected a particular pair of gametes (thus fixing the numerical identity of the future being) without any prior consideration of gene editing whatsoever; and (b) afterwards, but before IVF is carried out, we decide to use gene editing to remove a deleterious mutation from one of them. Here we are confronted with the question of whether this act is therapeutic in nature for the future human organism. Due to space considerations I will not engage with it. I want to thank an anonymous reviewer for flagging this issue.

^18^

Rulli, op. cit. note 1, p. 1078.

^19^

Some philosophers, such as Eric Olson and Ingmar Persson, have argued that human individuals were never early embryos, since early embryos can twin and lack structural integrity. Olson, E. T. (1999). *The human animal: Personal identity without psychology*. UK: Oxford University Press. Persson (1995), op. cit. note 13, p. 20. Although I do not have enough space to present a full defence of the position that early embryos are human individuals it is worth saying that both arguments fail. The twinning argument fails because if it were the case that things that have the potential to twin are not individuals then this would mean that amoebas and plants are not individuals, which is absurd. The lack of structural integrity argument (i.e. the mass of cells’ activities are not coordinated in the same way as the activities of an organism) fails since we have empirical evidence to the contrary, for example, which part of the early embryo will become the embryoblast and which will become the trophoblast seems to be determined by the point at which the sperm penetrated the oocyte. For a full defence that early embryos are human individuals see: Liao, S. M. (2010). Twinning, inorganic replacement, and the Organism View. *Ratio,* 23(1), 59–72. 10.1111/j.1467-9329.2009.00450.x; Liao, op. cit. note 7, pp. 334–350. It must be clear that different moral conclusions will follow depending on how we answer the two following questions: Are embryos persons? Do embryos have full moral status?

^20^

This is true even if the embryo twins afterwards.

^21^

Rulli, op. cit. note 1, p. 1079.

^22^

In a recent paper Giulia Cavaliere has defended this position: ‘if we are inclined to follow the second interpretation [CRISPR is not numerically identity affecting], then CRISPR is therapeutic as it pre‐emptively cures an embryo that will develop into a numerically identical child that does not have the genetic condition that is consciously avoided’. Cavaliere, op. cit. note 13, p. 220.

^23^

Huntington’s disease is a neurodegenerative disease of genetic origin. In it parts of the brain stop working properly over time. Symptoms of this disease usually appear in the third decade of life, and it is most of the times fatal after a period of up to 20 years.

^24^

Rulli, op. cit. note 1, p. 1079.

^25^

Ibid.

^26^

Ibid: 1080.

^27^

Ibid.

^28^

Parfit, op. cit. note 14.

^29^

Rulli, op. cit. note 1, p. 1079.

